# Type 1 diabetes associated with Hashimoto’s thyroiditis and juvenile rheumatoid arthritis : a case report

**DOI:** 10.1186/1546-0096-9-S1-P34

**Published:** 2011-09-14

**Authors:** Antonio Mellos, Angela Mauro, Milena Di Meglio, Carmela Granato, Laura Perrone, Alma N Olivieri

**Affiliations:** 1 Department of Pediatrics, Second University of Naples, Naples Italy

## Background

A girl of 11 years and two months came to our attention for diffuse arthralgias and lameness for about 6 months. This patient was affected by type 1 diabetes, which was debuted at the age of one year and 5 months [ICA weakly positive; GAD 0.1 AU/ml (n.v. <3); IA2 33 AU /ml (n.v. <1)], and she was also affected by Haschimoto’s thyroiditis since the age of 6 years and 6 months [ Ab anti –TG 21 IU/ml (n.v. < 100); Ab anti- TPO 230,4 IU/ml (n.v. <30)] . 9 months later, she began thyroid replacement therapy. Family history revealed that a maternal uncle suffered from diabetes mellitus by the age of 31 years; a maternal aunt suffered from diabetes and thyroiditis, too.

## Aim

We describe a case of polyarticular juvenile idiopathic arthritis, associated with diabetes and Haschimoto’s thyroiditis. This arthritis was particularly aggressive and our patient needed, after 17 months from the onset of arthritis, the addition of etanercept therapy with oral methotrexate and indomethacin. .

## Methods and results

Physical examination showed arthritis of the wrists, elbows, right knee, left ankle, right hip, first finger of the right hand, metacarpophalangeal joint of the third right and fifth finger left. Blood tests revealed inflammatory anemia ( hemoglobin 8.1 g / dl, ESR 125 mm /1 h, C-reactive protein 25.8 mg / dl, fibrinogen 682 mg / dl, serum iron 8 μg / dl, ferritin 210 ng/ ml ). Bone marrow aspirate was negative. Serology for rheumatoid factor (52 IU / ml) and antinuclear antibodies ( 1:160 ) was positive. Serological screen excluded celiac disease and connective tissue diseases. Ocular examination excluded iridocyclitis. Seropositive polyarticular type of juvenile rheumatoid arthritis was diagnosed. Arthritis was treated first with non-steroid anti-inflammatory agents [ naproxen (15 mg / kg / day), but only during the first two weeks after hospital discharge] and indomethacin ( dose variable between 1.25 mg/kg/day and 3 mg /kg / day) . Two weeks after hospital discharge, indomethacin was associated with to intramuscular methotrexate (10 mg / mÂ² / week). .

## Conclusions

Since the introduction of etanercept (0.4 mg / kg subcutaneously ,2 times/week), the functional limitation of joints affected by inflammation were progressively reduced and blood indices of disease activity have returned to normal range already after about 3 weeks.

## References

[B1] Nagy KH Lukacs K Sipos P Hermann R Madacsy L Soltesz G Type 1 Diabetes Associated with Hashimoto's thyroiditis and juvenile rheumatoid arthritis: a case report with clinical and genetic Investigations Pediatr Diabetes 2010 11 8 579 82 10.1111/j.1399-5448.2010.00676.x 21118342

